# Cytotoxicity of Fenugreek Sprout and Seed Extracts and Their Bioactive Constituents on MCF-7 Breast Cancer Cells

**DOI:** 10.3390/nu14040784

**Published:** 2022-02-13

**Authors:** Kholoud K. Khoja, Melanie-Jayne R. Howes, Robert Hider, Paul A. Sharp, Iain W. Farrell, Gladys O. Latunde-Dada

**Affiliations:** 1 Department of Nutritional Sciences, School of Life Course Sciences, King’s College London, Franklin-Wilkins-Building, 150 Stamford Street, London SE1 9NH, UK; kholoud.khoja@kcl.ac.uk (K.K.K.); paul.a.sharp@kcl.ac.uk (P.A.S.); 2 Royal Botanic Gardens Kew, Richmond TW9 3DS, UK; m.howes@kew.org (M.-J.R.H.); i.farrell@kew.org (I.W.F.); 3 Institute of Pharmaceutical Science, Faculty of Life Sciences and Medicine, King’s College London, Franklin-Wilkins-Building, 150 Stamford Street, London SE1 9NH, UK; robert.hider@kcl.ac.uk

**Keywords:** fenugreek, cytotoxicity, MCF-7, iron, cancer

## Abstract

*Trigonella foenum-graecum* L. (fenugreek), a member of the legume family (Fabaceae), is a promising source of bioactive phytochemicals, which explains its traditional use for a variety of metabolic disorders including cancer. The current study aimed to evaluate extracts of fenugreek seeds and sprouts, and some of their constituents, to compare their cytotoxic and antiproliferative activities in MCF-7 breast cancer cells. The extracts were chemically characterised using high-resolution accurate mass liquid chromatography-mass spectrometry to reveal the detection of compounds assigned as flavone *C*-glycosides including those derived from apigenin and luteolin, in addition to isoflavones. Five different flavones or their glycosides (apigenin, vicenin-2, vitexin, luteolin and orientin) and two isoflavones (daidzein and formononetin) were quantified in the fenugreek extracts. The 3-(4,5-dimethylthiazol-2-yl)-2,5-diphenyltetrazolium bromide assay using MCF-7 cells treated with fenugreek methanolic extracts showed dose- and time-dependent effects on cell viability. The MCF-7 cancer cells treated with the fenugreek methanolic extracts also displayed increased relative mitochondrial DNA damage as well as suppressed metastasis and proliferation. This study demonstrates the potential anti-cancer effects of fenugreek seeds and sprouts and reveals fenugreek sprouts as an untapped resource for bioactive compounds.

## 1. Introduction

*Trigonella foenum-graecum* L., commonly known as fenugreek, is an annual legume belonging to the Fabaceae family and is cultivated as a cash crop for its seeds. This herb is native to Afghanistan, Iran, Iraq, and Pakistan and has been introduced to a range of other countries [1]. The fenugreek seed extract is used as a flavouring ingredient to produce alternative syrups with maple or hydrolysed vegetable protein flavour [2]. It is also used as a tobacco flavouring, a perfume base, and a source of steroidal sapogenin compounds in the pharmaceutical industry [3]. Fenugreek leaves are commonly consumed as a vegetable and are a flavouring ingredient in Indian dishes, in which the plant is known by the common name ‘methi’ [2,4].

Fenugreek is of interest for use in cancer therapy because it has antioxidant and immunostimulatory activity [5]. Both its seeds and leaves contain phytochemicals including flavonoids, alkaloids, and tannins [4]. The putative therapeutic benefits of fenugreek are considered to be due to the phytoestrogens, saponins, and flavonoids present in the plant’s seeds and leaves [6]. Different types of flavonoids and their glycosides occur in fenugreek seed extracts including those derived from the flavones apigenin and luteolin, and the flavonol kaempferol [7]. In a previous study, fenugreek seed extracts fed to rats at a dose of 200 mg/kg body weight significantly inhibited 7,12-dimethylbenz(a)anthracene (DMBA)-induced mammary hyperplasia and decreased its incidence in these test animals [8], indicating that fenugreek seed extracts have potential anti-breast cancer benefits. Even though fenugreek leaves have long been used as a vegetable due to their nutritional benefits [2,4], there is limited data on their bioactive constituents. However, some studies suggest that germinated fenugreek seeds (i.e., sprouts) have a higher antioxidant content than boiled non-germinated fenugreek seeds [9]. The sprouting of fenugreek seeds is thought to increase the release, or the bioavailability, of bound antioxidants and other bioactive compounds [9]. Due to the promising but previously understudied effects of fenugreek sprouts, this study aimed to determine the bioactive compounds in fenugreek sprouts compared to seeds, and evaluate their effect on the proliferation of cultured breast cancer cells (MCF-7). Furthermore, the mechanism of apoptotic cell death induced by fenugreek in MCF-7 cells was also investigated to provide new insights into the potential for fenugreek sprouts to have a role against cancer.

## 2. Materials and Methods

### 2.1. Reagents and Chemicals

Unless otherwise stated, all the reagents and chemicals used in this study were purchased from Sigma-Aldrich Ltd. (Dorset, UK). Solutions of enzymes were all prepared freshly just before use.

### 2.2. Extraction

Organic fenugreek sprouts and seeds were obtained from Planet Organic (UK). The sprouts were air-dried at room temperature, and both the seeds and sprouts were then ground separately in a classic Moulinex AR1043 grinder prior to sequential Soxhlet extraction, described as follows.

A total of 100 g of milled powder from the dried sprouts or seeds was extracted with 200 mL methanol [MeOH], (Sigma 34860) *n*-hexane (Sigma 139386), or chloroform (Sigma 650498) for 24 h at 40 ± 2 °C. The organic solvent was 10 times (*v/w*) the sample weight, and extraction via Soxhlet continued until the solvent became colourless. The solvents were used in the following order: (A) MeOH/*n*-hexane; extract residue then extracted with chloroform; (B) chloroform/*n*-hexane; extract residue then extracted with MeOH; (C) chloroform only. The extracts residues were stored at −20 °C prior to analysis.

### 2.3. High-Resolution Accurate Mass Liquid Chromatography-Mass Spectrometry Analysis of Extracts

The fenugreek extracts were subjected to high-resolution accurate mass liquid chromatography-photodiode array-mass spectrometry (HRAM LC-PDA-MS/MS) analysis. The extracts were prepared at 10 mg/mL in 100% MeOH and then sonicated for 20 min before LC-MS analysis. The analyses were performed on a Thermo Scientific system consisting of an ‘Accela’ U-HPLC unit with a photodiode array detector and an ‘LTQ Orbitrap XL’ mass spectrometer fitted with an electrospray source (Thermo Scientific, Waltham, MA, USA). Chromatography was performed with 5 µL sample injections (per extract) onto a 150 mm × 3 mm, 3 µm Luna C-18 column (Phenomenex, Torrance, CA, USA) using the following 400 µL/min mobile phase gradient of CH_3_OH/H_2_O/CH_3_CN + 1% HCOOH: 0:90:10 (0 min), 90:0:10 (60 min), 90:0:10 (65 min), 0:90:10 (67 min), and 0:90:10 (70 min). The electrospray source was set to record high-resolution (30 k resolution) mass spectra [MS1] (*m/z* 125–2000) in positive mode using the orbitrap and data-dependent MS2 and MS3 spectra in both modes using the linear ion trap. Detected compounds were assigned by comparison of the accurate mass data and interpretation of the MS2, MS3, and UV spectra and the published compound assignment system [10].

### 2.4. Quantitative Analysis of Compounds Detected in Fenugreek Extracts

The fenugreek sprout and seed extracts were analysed at 50 mg/mL in duplicate using a Thermo Fisher Velos Pro LC-PDA_MS. Samples (5 μL) were injected directly onto a Phenomenex Luna C-18 (2) column (150 mm, 3 mm i.d., 3 μm particle size) at 400 μL/min and eluted using a linear gradient of 90:0:10 (t = 0 min) to 0:90:10 (t = 20–25 min), returning to 90:0:10 (t = 27–30 min). The solvents were water, MeOH, and 1% formic acid in acetonitrile, respectively. The column was maintained at 30 °C. Compounds were detected with a Thermo Fisher Velos Pro Dual-Pressure Linear Ion Trap Mass Spectrometer. Samples were scanned using the ITMS from *m/z* 200 to 600, and the UV peak areas were quantified against the calibration curves of the reference standards (apigenin, vicenin-2, vitexin, luteolin, orientin, formononetin, and daidzein; Sigma), which were analysed in duplicate over a concentration range of 0.32–2000 µg/mL using the same LC–PDA-MS method.

### 2.5. Cell Culture

The breast cancer cell line MCF-7 cells on passages 35–40 and non-tumorigenic epithelial cell line MCF-10A cells on passages 4–9 were obtained from the American Type Culture Collection [ATCC].

The cells were sub-cultured in a 75-cm^2^ flask at ~70% confluency. The growth medium contained Dulbecco’s modified Eagle medium [DMEM] and high glucose with glutamine (Sigma D5796), and was supplemented with 10% foetal bovine serum [FBS] (Sigma 2442) and 1% penicillin-streptomycin (Sigma 9644). The MCF-10A cells were sub-cultured in DMEM (Sigma 5648) and Ham’s F-12 base (Sigma D9785) supplemented with 20 ng/mL epidermal growth factor (Sigma E9644), 100 ng/mL cholera toxin (Sigma C8052), 0.01 mg/mL human insulin (Sigma I2643), 500 ng/mL hydrocortisone, and 5% horse serum (Gibco 26050-088).

Both cell types were incubated at 37 °C, with 95% humidity and 5% carbon dioxide. The growth medium was changed every two days. The cells were harvested after three washes with phosphate-buffered saline [PBS] (Sigma P5493) and incubated with 2–5 mL Trypsin–ethylenediaminetetraacetic acid [EDTA] (Sigma T3924) until the cells detached (5–10 min). Culture medium (5 mL) was added, and the cells were collected and centrifuged at 107× *g* for five minutes.

### 2.6. Cell Viability Studies

Cells were seeded at a density of 1 × 10^4^ cells/cm^2^ in 96-well plates. The medium was discarded, and the cells were washed three times with sterile phosphate-buffered saline [PBS] and then incubated and treated at different time points according to the experimental plan. The cells were treated with different concentrations of the test compounds (fenugreek sprouts, fenugreek seeds) and incubated for a variable time. Following this, 100 µL of fresh modified Eagle’s medium [DME] (no phenol red Thermofisher 210633029) along with 10 µL of dimethylthiazol-2-yl)-2,5-diphenyltetrazolium bromide [MTT] (Sigma M2003) sterile solution (5 mg/mL MTT in PBS) were added to each well. After incubating for 3 h in the dark at 37 °C, 100 μL of a solubilisation buffer in dimethyl sulfoxide [DMSO] (Sigma D4540) was added and incubated for 15 min at room temperature. To determine the MTT reaction in the cells, optical density was read in a microplate reader (Bio-Tek ELx800) at 490 nm. Cell viability was expressed as a percentage of the controls. The calculation was conducted as outlined below:

50% inhibition = IC_50_ = 100/(1 + 10^((LogIC50-X)*HillSlope)).

### 2.7. Proliferation Assay

This assay quantifies cell proliferation based on the incorporation of the pyrimidine analogue 5-bromo-2′-deoxyuridine [BrdU] into cellular DNA (during DNA replication) in exchange for thymidine. The integrated BrdU is detected by the peroxidase-conjugated anti-BrdU antibody, followed by a colourimetric reaction including tetramethylbenzidine [TMB].

Herein, the assay was used according to the manufacturer’s instructions. The Cell Proliferation enzyme-linked immunosorbent assay [ELISA], BrdU colourimetric (Roche, 11647229001) Assay Kit was used according to the manufacturer’s instructions. The treated cells were seeded at 10^4^ in a white-rimmed 96-well plate (Corning 353377). The BrdU was added to a final concentration of 10 µM at 10 µL BrdU/100 µL sterile DME per well for one hour at 37 °C. After removing the labelling medium, the plates were incubated for 30 min at room temperature to fix the cells, and the DNA was denatured after adding 200 mL/well FixDenat. The cells were incubated with 50 µL/well of peroxidase-conjugated anti-BrdU antibody for 90 min at room temperature after the removal of the FixDenat solution. Immune complexes were detected after washing with PBS once using 100 µL/well TMB substrate reaction until colour development. Luminescence was measured after an incubation of 10–15 min at 450 nm using a Monolight 3096 microplate luminometer (BD Bioscience). The MCF-7 cells were treated overnight with the IC_50_ of the finished sprouts and seeds in methanol extracts.

### 2.8. Real-Time Quantitative PCR

Total genomic DNA was extracted using Qiagen (Germany) according to the manufacturer’s instructions. DNA samples were sonicated as described [11], and the concentration was determined using the NanoDrop and adjusted to 10 ng/μL. The Applied BioSystem Fast Real-Time PCR System-7900HT Kit was used to carry out the qRT-PCR of DNA of the samples with specific forward and reverse primers and probes (Roche, Germany) designed to target mitochondrial DNA using the human mitochondrial genome [hMito] gene; the level was normalised to the human Beta-2 microglobulin [hB2M] nuclear gene. Following this, the samples were seeded into 96- × 4-well PCR plates using a robot (BioMex FX-Beckman Coulter). The results of relative content were expressed as ∆Ct = Ct (gene of interest) − Ct (housekeeping gene) and ∆∆Ct = ∆Ct (treated) − ∆Ct (control).

### 2.9. Statistical Analysis

Experiments were performed in 3–6 replicates, and data are shown as mean ± standard error of the means (SEM). The comparisons were analysed using one-way or two-way analysis of variance [ANOVA] followed by Tukey’s post hoc test where appropriate using the GraphPad Prism software. The significance level was at *p* ≤ 0.05.

## 3. Results

### 3.1. Fenugreek Sprout and Seed Extractions

The extract yields from the dry ground fenugreek sprouts and seeds after Soxhlet extraction are shown in Table 1 with different yields being observed between seeds and sprouts and the extraction solvent.

### 3.2. LC–MS Analysis of Fenugreek Seed and Sprout Extracts

The assigned compounds detected in the fenugreek extracts are shown in Supplementary Table S1. Numerous apigenin *C*-glycosides including compounds assigned as vitexin and isovitexin were the main compound class detected in the first FPME, third FPME, first FSME, and third FSME. Detected also in the first and third FPMEs and FSMEs were compounds assigned as luteolin *C*-glycosides.

Other compound classes detected included those assigned as isoflavones: 3′,5,7-trihydroxy-5′-methoxy-isoflavone in the first and third FPMEs and third FPHE, formononetin hexoside in the second FPCE (after hexane, before methanol) and daidzein or isomer in the first and third FPMEs. A range of compounds assigned as steroidal saponins was also detected primarily in the methanolic fenugreek extracts.

### 3.3. Content of Specific Flavonoids in the Fenugreek Extracts

Certain flavonoids that were detected in the fenugreek extracts or the aglycones of the detected flavonoids (Supplementary Table S1) were sourced as reference standards for quantification in the fenugreek methanol extracts and for testing in the MTT assay on MCF-7 cells (Table 2).

The quantitative analyses revealed that certain flavonoids (ascertained as bioactive; Table 1), except for formononetin, occurred at higher concentrations in the third FPME compared to the first. Moreover, orientin and daidzein occurred at higher concentrations in the first FSME, while vicenin-2 and vitexin occurred at higher concentrations in the third. The IC_50_ values for apigenin (39.91 µM), luteolin (35 µM), and formononetin (50.13 µM) indicated these flavonoids could reduce the cell viability of MCF-7 cells (Table 2), indicating these compounds could have contributed to the activity of the extracts in which they were detected.

### 3.4. Cell Viability

**Figure 1:**
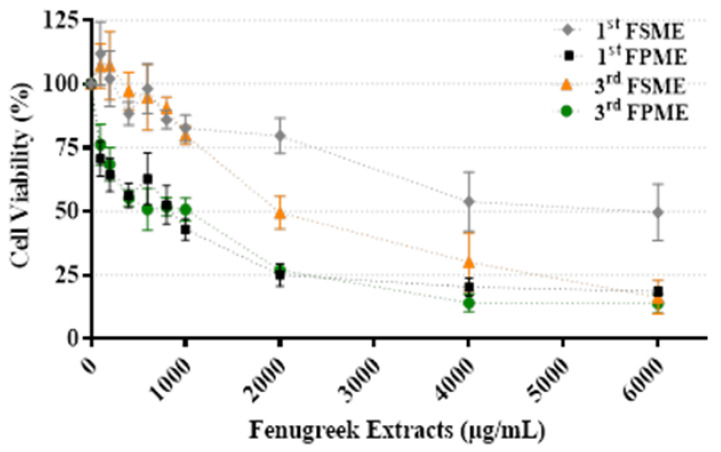
Cell viability of MCF-7 cells treated with various concentrations of fenugreek sprout and seed methanol extracts (0–6000 µg/mL) for 24 h. 1st Fenugreek Sprouts Methanol Extraction [1st FPME], 3rd Fenugreek Sprouts Methanol Extraction [3rd FPME], 1st Fenugreek Seeds Methanol Extraction [1st FSME], 3rd Fenugreek Seeds Methanol Extraction [3rd FSME]. The values are presented as means *n* = 6 ± SEM.

Figure 1 presents the viability of the MCF-7 cells treated with the fenugreek methanol extracts; data are presented as a percentage and normalised against the untreated control. Following 24 h of incubation, all fenugreek methanol extracts reduced the cellular viability of the cultured breast cancer MCF-7 cells tested over the concentration range (0–6000 µg/mL; Figure 1). The first and third methanol extracts prepared from fenugreek seeds and sprouts produced IC_50_ values of 5605 and 812 µg/mL and 1400 and 526 µg/mL, respectively. The cell viability results demonstrated that both MeOH extracts from the fenugreek sprouts were more potent than those from the seeds. For both seeds and sprouts, the third MeOH extracts were more potent than the first. Thus, the third MeOH fenugreek extracts were selected for further study.

**Figure 2:**
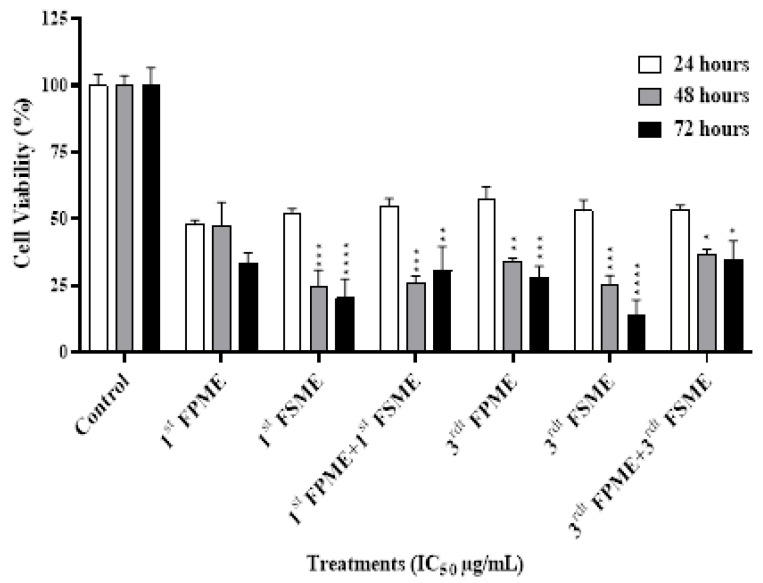
Fenugreek-inhibited cell growth in MCF-7 human breast cancer cells. The viability of the cells was analysed using cell MTT assays. 1st Fenugreek Sprouts Methanol Extraction [1st FPME], 3rd Fenugreek Sprouts Methanol Extraction [3rd FPME], 1st Fenugreek Seeds Methanol Extraction [1st FSME], 3rd Fenugreek Seeds Methanol Extraction [3rd FSME]. The IC_50_ treatments of the first FSME at 5605 µg/mL, first FPME at 812 µg/mL, third FSME at 1400 µg/mL, and third FPME at 526 µg/mL for 24, 48, and 72 h decreased the viability of the MCF-7 cells in a time-dependent manner. The values are presented as means *n* = 6 ± SEM. The data were analysed using a one-way ANOVA test. Different asterisks indicate significant differences between data in the same group (* *p* ≤ 0.05, ** *p* ≤ 0.01, *** *p* ≤ 0.001, and **** *p* ≤ 0.0001).

The cytotoxic effect of the fenugreek methanol extracts on the MCF-7 cells was evaluated using an MTT assay. As shown in Figure 2, the cell viability percentages of the MCF-7 cells were 47.7, 47.3, 33.52% and 47.7, 47.3, 33.5% with the first, and 53.2, 25.4, 13.8% and 57.71, 34.22, 28.27% with the third methanol seed and sprout extracts, respectively, which caused a time-dependent inhibition of the MCF-7 cell growth (over 24, 48, and 72 h). At 24 h, the IC_50_ value was significantly higher than at 48 and 72 h of incubation time, except for the cells treated with the first FSME.

### 3.5. Cell Proliferation

**Figure 3:**
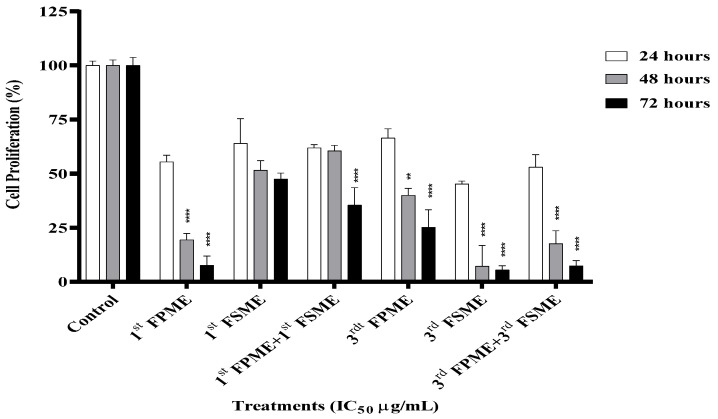
Fenugreek-inhibited cell proliferation of MCF-7 human breast cancer cells. The proliferation of cells was analysed using a cell proliferation assay. 1st Fenugreek Sprouts Methanol Extraction [1st FPME], 3rd Fenugreek Sprouts Methanol Extraction [3rd FPME], 1st Fenugreek Seeds Methanol Extraction [1st FSME], 3rd Fenugreek Seeds Methanol Extraction [3rd FSME]. The IC_50_ treatment of the 3rd FSME at 5605 µg/mL, 1st FPME at 812 µg/mL, 3rd FSME at 1400 µg/mL, and 3rd FPME at 526 µg/mL for 24, 48, and 72 h decreased the proliferation of the MCF-7 cells in a time-dependent manner. The values are presented as means *n* = 6 ± SEM. The data were analysed using a one-way ANOVA test. Different asterisks indicate significant differences between data in the same group (** *p* ≤ 0.01, and **** *p* ≤ 0.0001).

A proliferation assay was performed on the MCF-7 cells following the various treatments. The fenugreek methanol extracts inhibited cell proliferation in a time-dependent manner (Figure 3). The IC_50_ values of the first and third methanol extracts from the seeds and sprouts at 5605 and 812 µg/mL and 1400 and 526 µg/mL, respectively, decreased the proliferation of the MCF-7 cells significantly (*p* ≤ 0.0001) after 48 and 72 h. However, the first FSME treatment did not affect the cells’ DNA proliferation over the concentration range tested (Figure 3).

### 3.6. Mitochondrial DNA (mtDNA) Damage

**Figure 4:**
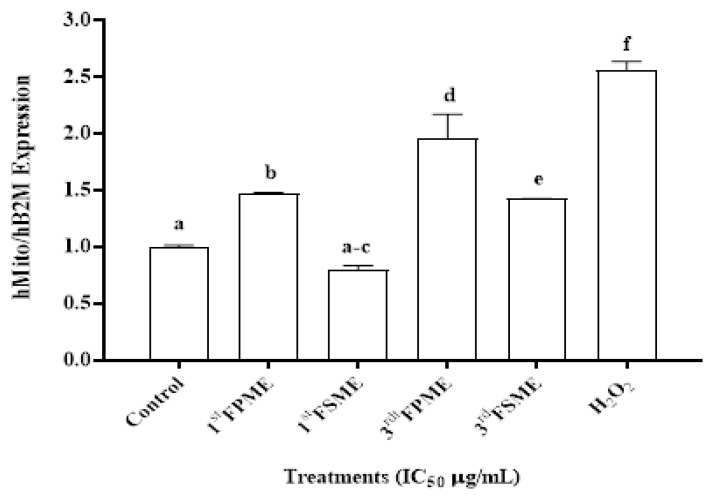
The mtDNA relative content in the MCF-7 cells. Amplified by RT-PCR and normalised to the human Beta-2 microglobulin [hB2M] nuclear gene after IC_50_ treatment of the 3rd FSME at 5605 µg/mL, 1st FPME at 812 µg/mL, 3rd FSME at 1400 µg/mL, and 3rd FPME at 526 µg/mL in the MCF-7 cells for 24 h; H_2_O_2_ was used as a positive control. 1st Fenugreek Sprouts Methanol Extraction [1st FPME], 3rd Fenugreek Sprouts Methanol Extraction [3rd FPME], 1st Fenugreek Seeds Methanol Extraction [1st FSME], 3rd Fenugreek Seeds Methanol Extraction [3rd FSME], hydrogen peroxide [H_2_O_2_] (Sigma H1009). The values are presented as means *n* = 4 ± SEM. The data were analysed using a two-way ANOVA test. The different letters on top of the bars indicate significant differences (*p* ≤ 0.05) between the data.

The third fenugreek seeds sprout methanol extracts significantly promoted cell mtDNA damage (*p* ≤ 0.05), while the sprout extract was more potent than the seed extract overall. The increased relative mtDNA damage inhibited the standard characteristics of metastatic MCF-7 cell growth and proliferation (Figure 4), and this could have contributed to the high cell death of the MCF-7 cells that were treated with the fenugreek extracts.

## 4. Discussion

Of the three extraction solvents used (*n*-hexane, methanol, and chloroform), methanol was the most efficient at obtaining the highest extraction yields from the fenugreek sprouts and seeds. This was also demonstrated in a recent study in which bioactive phytochemicals were extracted from fenugreek seeds using a hydromethanolic solution (50/50, *v/v*) [7]. LC-MS analysis revealed that flavonoids and their glycosides, particularly apigenin *C*-glycosides, were the main compounds detected in the fenugreek extracts. Luteolin *C*-glycosides were also detected but were less abundant. This was consistent with a recent study [12] in which apigenin and luteolin adducts were the most abundant flavonoid glycosides in germinated fenugreek seed extracts. In an earlier study [7], the major constituents included non-acylated flavonoid glycosides (48%), acylated flavonoid glycosides (46%), and phenolic acids (6%), and the major flavonoid constituents included apigenin (61%), luteolin (21%), and kaempferol (11%). These results were consistent with those of the current study, in which flavone derivatives were predominant in the fenugreek seed extracts.

Herein, five different flavones or their glycosides (apigenin, vicenin-2, vitexin, luteolin and orientin) and two isoflavones (daidzein and formononetin) were quantified in the fenugreek extracts. Apigenin glycosides including vitexin and vicenin-1 and -2 were previously quantified in fenugreek seeds [13]. Among the flavones or their glycosides detected, apigenin was more abundant in the FPMEs than in the FSMEs, while the yields of vicenin-2, luteolin, orientin, and vitexin were more abundant in the FSMEs than in the FPMEs. Among the isoflavones detected, daidzein was more abundant in the seeds, while formononetin was more abundant in the sprouts. Thus, consistent with the literature [13,14], the findings from the current study indicated that flavones are primarily present in fenugreek seeds, while isoflavones are more abundant in fenugreek sprouts.

Herein, the FPMEs and FSMEs reduced cell viability in a dose- and time-dependent manner, consistent with two previous studies on seeds [15,16], and showed these effects for the first time with fenugreek sprout extracts. Sebastian and Thampan [16] reported that an ethanolic extract of fenugreek seeds at a concentration of 50 µg/mL resulted in a 70% decrease in MCF-7 cell viability during a 72-h culture [16]. Ahmed et al. [15] reported the IC_50_ values of FSMEs against MCF-7 cell lines to be in the range of 2850–3140 µg/mL. Meanwhile, the IC_50_ values determined by [15] were in the range of 1400–5605 µg/mL, as similarly observed in the current study. However, the processing techniques of fenugreek seeds have been shown to affect the nutritional/chemical profile and antioxidant activity of fenugreek extracts [17].

The MTT assay in the current study showed that the first and third FPMEs had IC_50_ values of 812 and 526 µg/mL, respectively, and were significantly more potent than the first and third FSMEs, which had IC_50_ values of 5605 and 1400 µg/mL, respectively. These findings present new data to indicate that fenugreek sprouts have higher concentrations of cytotoxic compounds. In these extracts, the compounds detected included isoflavones, assigned as daidzein and formononetin, and were mainly present in the sprouts, which exhibited higher IC_50_ values of 152.2 µM and 50.13 µM, respectively. These phytochemicals may have contributed to the observed cytotoxicity. This was consistent with earlier findings reporting that formononetin, a phytoestrogen, induced apoptotic cell death in diverse types of human cancers [18]. Moreover, soybean isoflavone (daidzein) has been shown to be cytotoxic to cultures of MCF-7 and MDA-MB-231 breast cancer cells [19]. Furthermore, we also report the detection of compounds assigned as steroidal saponins in the sprout extracts and emphasise that these and other detected compound classes in the fenugreek extracts may also have contributed to the observed cytotoxic effects in the current study. The findings from this study, therefore, underpin future research to elucidate which other chemical constituents of fenugreek sprouts mediate potential anti-cancer effects.

The anti-cancer activity of the fenugreek extracts was also evaluated via the degree of induced mtDNA damage. Mitochondrial dysfunction underlies the pathogenesis of a variety of disorders including cancer [20,21]. The survival of cancer cells is directly related to mtDNA half-life; therefore, an effective anti-cancer bioactive compound can significantly promote mtDNA damage (i.e., shorten its half-life). In the current study, the metastatic MCF-7 cells treated with fenugreek seed extracts exhibited significantly increased mtDNA damage (determined by the relative levels), which resulted in the suppression of cell proliferation, and hence suppressed the metastatic potential of the cells.

While there have been numerous published studies on the chemistry and bioactivities of fenugreek seeds [21,22,23,24,25], fenugreek sprouts and constituents, and their effects to mediate changes in cell viability and cell proliferation in MCF-7 cells, have not been assessed previously, which we present here for the first time. In conclusion, this study revealed that chemically profiled fenugreek seed and sprout extracts show anti-cancer effects in vitro, and provides the first evidence for the untapped potential of fenugreek sprouts for their role against certain forms of cancer.

## Figures and Tables

**Table 1:** Extraction yields from fenugreek sprouts and seeds using different solvents.

Sample	Organic Solvent	Sample (g)	Yield (%)
Sprouts	1st Methanol Extraction	100	22.83
	2nd Chloroform Extraction		1.88
	3rd Hexane Extraction		0.09
	1st Hexane Extraction	100	6.14
	2nd Chloroform Extraction		1.24
	3rd Methanol Extraction		12.51
	Chloroform Extraction	100	11.09
Seeds	1st Methanol Extraction	100	17.19
	2nd Chloroform Extraction		1.25
	3rd Hexane Extraction		0.00
	1st Hexane Extraction	100	5.93
	2nd Chloroform Extraction		1.45
	3rd Methanol Extraction		10.95
	Chloroform Extraction	100	3.08

**Table 2:** Flavonoids quantified in the fenugreek methanol extracts and their IC_50_ values when tested individually on MCF-7 cells.

Plant Part and Extract (Assigned Compound)			Synonym	Molecular Weight	Molecular Formula	IC_50_ µM	1st FPME µg/50 mg	3rd FPME µg/50 mg	1st FSME µg/50 mg	3rd FSME µg/50 mg
Flavones		Apigenin	4′,5,7-Trihydroxyflavone	270.24	C_15_H_10_O_5_	39.91 ± 1.72	6.3 [0.023]	9.84 [0.036]	0.78 [0.002]	0.62 [0.002]
		Vicenin-2	Apigenin 6,8-*C*-di-glucoside	594.52	C_27_H_30_O_15_	199.1 ± 1.48	10.0 [0.017]	12.0 [0.021]	30.0 [0.050]	279.0 [0.470]
		Vitexin	Apigenin 8-*C*-glucoside	432.38	C_21_H_20_O_10_	247.4 ± 1.61	8.5 [0.020]	11.5 [0.027]	57.8114.6 [0.266]	121.4 [0.281
		Luteolin	3′,4′,5,7-Tetrahydroxyflavone	286.24	C_15_H_10_O_6_	35 ± 1.95	13.0 [0.045]	14.4 [0.050]	32.6 [0.114]	28.6 [0.100]
		Orientin	Luteolin 8-*C*- glucoside	448.38	C_21_H_20_O_11_	277 ± 2.95	63.0 [0.141]	73.0 [0.163]	5212.0 [11.632]	11538.0 [25.755]
Isoflavones		Daidzein	4′,7-Dihydroxyisoflavone	254.24	C_15_H_10_O_4_	152.2 ± 1.94	3.8 [0.015]	3.6 [0.014]	127.8 [0.504]	12.5 [0.049]
		Formononetin	Daidzein 4′-methyl ether	268.26	C_16_H_12_O_4_	50.13 ± 2.52	34.0 [0.127]	16.1 [0.060]	5.2 [0.020]	2.9 [0.011]

Flavonoid content of fenugreek sprout and seed extracts (µg/50 mg extract), [mmol/50 mg extract]. 1st Fenugreek Sprouts Methanol Extraction [1st FPME], 3rd Fenugreek Sprouts Methanol Extraction [3rd FPME], 1st Fenugreek Seeds Methanol Extraction [1st FSME], 3rd Fenugreek Seeds Methanol Extraction [3rd FSME]. The values in parentheses are in the 95% range. The IC_50_ values for the individual flavonoids on MCF-7 cell death at 24 h. The values are presented as means *n* = 3 ± SEM.

## Supplementary Materials

The following supplementary materials can be downloaded at: https://www.mdpi.com/article/10.3390/nu14040784/s1, Table S1: Detected compounds assigned from LC–MS analysis of the *Trigonella foenum-graecum* extracts.
