# Supplementary material for: Cytotoxicity of Fenugreek Sprout and Seed Extracts and Their Bioactive Constituents on MCF-7 Breast Cancer Cells
Source: Nutrients. 2022 Feb 13;14(4):784. doi: 10.3390/nu14040784 (PMC8879394; doi:10.3390/nu14040784)
Supplement: Supplementary file 1 [file nutrients-14-00784-s001.zip › nutrients-1551641 SI.pdf]

Supplementary Information

Table S1. Detected compounds assigned from LC–MS analysis of the *Trigonella foenum-graecum* extracts.

Assigned compound# (or isomer)	Retention time (min)	Molecular formula	Experimental (*m*/*z*)	ppm	Ion	1st FSME ppm#	3rd FSME ppm#	1st FPME ppm#	3rd FPME ppm#	1st FSHE ppm#	1st FPHE ppm#	3rd FPHE ppm#	2nd FSCE (after hexane, before methanol) ppm#	2nd FPCE (after hexane, before methanol) ppm#	2nd FSCE (after methanol before hexane) ppm#	2nd FPCE (after methanol before hexane) ppm#
Tyrosine	2.6	$C_9H_{11}NO_3$	182.0809	1.4	$[M + H]^+$	1.701	Nd	Nd	Nd	Nd	Nd	Nd	Nd	Nd	Nd	Nd
Glutamyltyrosine	3.0	$C_{14}H_{18}N_2O_6$	311.1231	2.1	$[M + H]^+$	2.002	2.098	Nd	Nd	Nd	Nd	Nd	Nd	Nd	Nd	Nd
Phenylalanine	3.0	$C_9H_{11}NO_2$	166.0855	4.5	$[M + H]^+$	2.259	2.560	4.607	4.788	Nd	Nd	3.222	Nd	Nd	Nd	2.861
Tryptophan	4.9	$C_{11}H_{12}N_2O_2$	205.0969	1.2	$[M + H]^+$	1.483	2.068	2.263	3.433	Nd	Nd	4.019	Nd	Nd	Nd	Nd
Glutamyl-phenylalanine	7.0	$C_{14}H_{18}N_2O_5$	295.1284	1.5	$[M + H]^+$	1.146	1.552	1.146	1.552	Nd	Nd	Nd	Nd	Nd	Nd	Nd
Methyltryptophan	9.2	$C_{12}H_{14}N_2O_2$	219.1124	1.8	$[M + H]^+$	Nd	Nd	1.160	1.115	Nd	Nd	Nd	Nd	Nd	Nd	Nd
6,8-Di-*C*-hexosyl-apigenin (Vicenin-2 or isomer)	9.7	$C_{27}H_{30}O_{15}$	595.1652	0.9	$[M + H]^+$	0.885	0.263	Nd	Nd	Nd	Nd	Nd	Nd	Nd	Nd	Nd
6,8-Di-*C*-hexosyl-apigenin (Vicenin-2 or isomer)	10.5	$C_{27}H_{30}O_{15}$	595.1652	0.9	$[M + H]^+$	2.313	2.111	0.263	1.086	Nd	Nd	Nd	Nd	Nd	Nd	Nd
C-Pentosyl-*C*-hexosylapigenin*	10.7	$C_{26}H_{28}O_{14}$	565.1542	1.7	$[M + H]^+$	1.667	1.030	0.923	0.923	Nd	Nd	Nd	Nd	Nd	Nd	Nd
C-Pentosyl-*C*-hexosylapigenin*	11.2	$C_{26}H_{28}O_{14}$	565.1543	1.7	$[M + H]^+$	1.560	1.450	1.136	0.923	Nd	Nd	Nd	Nd	Nd	Nd	Nd
C-Pentosyl-*C*-hexosylapigenin*	11.3	$C_{26}H_{28}O_{14}$	565.1543	1.7	$[M + H]^+$	1.560	2.109	1.454	0.375	Nd	Nd	Nd	Nd	Nd	Nd	Nd
C-Pentosyl-*C*-hexosylapigenin*	11.6	$C_{26}H_{28}O_{14}$	565.1548	0.7	$[M + H]^+$	1.242	0.269	2.321	1.030	Nd	Nd	Nd	Nd	Nd	Nd	Nd
C-Pentosyl-*C*-hexosylapigenin*	12.7	$C_{26}H_{28}O_{14}$	565.1540	2.1	$[M + H]^+$	2.109	2.427	2.976	3.401	Nd	Nd	Nd	Nd	Nd	Nd	Nd
C-Pentosyl-*C*-hexosylapigenin*	13.6	$C_{26}H_{28}O_{14}$	565.1540	2.1	$[M + H]^+$	2.109	2.534	3.188	2.427	Nd	Nd	Nd	Nd	Nd	1.242	Nd
Isoorientin	13.9	$C_{21}H_{20}O_{11}$	449.1067	2.5	$[M + H]^+$	2.489	1.064	Nd	0.797	Nd	Nd	Nd	Nd	Nd	1.465	Nd
Orientin	14.3	$C_{21}H_{20}O_{11}$	449.1066	2.7	$[M + H]^+$	2.756	1.665	Nd	0.530	Nd	Nd	Nd	Nd	Nd	0.797	Nd
C-Pentosyl-*C*-hexosylapigenin*	14.9	$C_{26}H_{28}O_{14}$	565.1545	1.2	$[M + H]^+$	1.242	1.348	1.242	2.321	Nd	Nd	Nd	Nd	Nd	Nd	Nd
C-Pentosyl-*C*-hexosylapigenin*	15.3	$C_{26}H_{28}O_{14}$	565.1543	1.6	$[M + H]^+$	1.560	1.560	0.587	2.109	Nd	Nd	Nd	Nd	Nd	Nd	Nd

C-Pentosyl-*C*-hexosylapigenin*	16.0	$C_{26}H_{28}O_{14}$	565.1543	1.6	$[M + H]^+$	1.560	0.817	1.667	1.242	Nd	Nd	Nd	Nd	Nd	Nd	Nd
Vitexin	16.7	$C_{21}H_{20}O_{10}$	433.1117	2.8	$[M + H]^+$	2.778	2.201	1.855	2.132	Nd	Nd	Nd	Nd	Nd	1.924	Nd
C-Pentosyl-*C*-pentosylapigenin	16.9	$C_{25}H_{26}O_{13}$	535.1437	1.7	$[M + H]^+$	1.751	1.994	2.106	0.966	Nd	Nd	2.331	Nd	Nd	Nd	Nd
Isovitexin	17.5	$C_{21}H_{20}O_{10}$	433.1116	3.1	$[M + H]^+$	3.124	1.577	2.062	1.785	Nd	Nd	1.855	Nd	Nd	2.616	Nd
C-Pentosyl-*C*-pentosylapigenin	17.9	$C_{25}H_{26}O_{13}$	535.1437	1.7	$[M + H]^+$	1.751	2.106	1.639	0.836	Nd	Nd	Nd	Nd	Nd	Nd	Nd
C-Pentosyl-*C*-pentosylapigenin	18.4	$C_{25}H_{26}O_{13}$	535.1439	1.3	$[M + H]^+$	1.415	1.191	0.611	1.639	Nd	Nd	Nd	Nd	Nd	Nd	Nd
Hydroxyluteolin hexoside	18.5	$C_{21}H_{20}O_{12}$	465.1020	1.6	$[M + H]^+$	Nd	Nd	0.521	0.005	Nd	Nd	Nd	Nd	Nd	Nd	Nd
C-Pentosyl-*C*-pentosylapigenin	19.3	$C_{25}H_{26}O_{13}$	535.1429	3.2	$[M + H]^+$	3.134	3.003	1.415	2.331	Nd	Nd	Nd	Nd	Nd	Nd	Nd
C-Pentosyl-*C*-pentosylapigenin	19.9	$C_{25}H_{26}O_{13}$	535.1437	1.7	$[M + H]^+$	1.639	0.836	0.724	2.443	Nd	Nd	Nd	Nd	Nd	Nd	Nd
Hexanol *O*-pentosyl-hexoside	19.9	$C_{17}H_{32}O_{10}$	414.2331	0.7	$[M+NH_4]^+$	Nd	Nd	1.189	0.682	Nd	Nd	3.097	Nd	Nd	Nd	Nd
8-*C*-Galactosyl-6-*C*-quinovosyl-apigenin	20.5	$C_{27}H_{30}O_{14}$	579.1696	2.1	$[M + H]^+$	2.196	1.471	1.575	1.885	Nd	Nd	Nd	Nd	Nd	Nd	Nd
C-Pentosyl-*C*-pentosylapigenin	20.8	$C_{25}H_{26}O_{13}$	535.1436	1.9	$[M + H]^+$	1.863	3.134	0.611	0.173	Nd	Nd	Nd	Nd	Nd	Nd	Nd
3′,5,7-Trihydroxy-5′-methoxy-isoflavone	21.8	$C_{16}H_{12}O_6$	301.0706	0.2	$[M + H]^+$	Nd	Nd	1.377	1.477	Nd	Nd	1.277	Nd	Nd	Nd	Nd
Apigenin *O*-hexuronide	22.4	$C_{21}H_{18}O_{11}$	447.0919	0.7	$[M + H]^+$	Nd	Nd	0.733	1.762	Nd	Nd	Nd	Nd	Nd	Nd	Nd
Apigenin rhamnosyl-diglucuronide	23.4	$C_{36}H_{32}O_{19}$	769.1603	1.0	$[M + H]^+$	Nd	Nd	0.371	0.930	Nd	Nd	Nd	Nd	Nd	Nd	Nd
Apigenin rhamnosyl-pentoside	23.9	$C_{26}H_{28}O_{13}$	549.1599	0.7	$[M + H]^+$	0.760	1.197	Nd	Nd	Nd	Nd	Nd	Nd	Nd	Nd	Nd
Hydroxy-methoxy-isoflavone hexoside (formononetin hexoside or isomer)	24.0	$C_{22}H_{22}O_9$	431.1328	2.0	$[M + H]^+$	Nd	Nd	1.969	2.316	Nd	Nd	3.035	Nd	2.456	Nd	1.829
2′′-*O*-*p*-Coumaroyl-orientin	24.7	$C_{30}H_{26}O_{13}$	595.1434	2.1	$[M + H]^+$	1.995	2.297	Nd	Nd	Nd	Nd	Nd	Nd	Nd	2.096	Nd
Eurycomaoside	25.2	$C_{25}H_{38}O_{12}$	548.2694	1.4	$[M+NH_4]^+$	1.025	1.463	Nd	Nd	Nd	Nd	Nd	Nd	Nd	Nd	Nd
Apigenin hexoside	25.5	$C_{21}H_{20}O_{10}$	433.1125	1.0	$[M + H]^+$	1.070	0.100	Nd	Nd	Nd	Nd	Nd	Nd	Nd	Nd	Nd
Apigenin 4'-*O*-[4-hydroxy-3-methoxy-*E*-cinnamoyl-glucuronosyl-glucuronoside]	25.7	$C_{37}H_{34}O_{20}$	799.1712	0.5	$[M + H]^+$	Nd	Nd	0.525	0.525	Nd	Nd	Nd	Nd	Nd	Nd	Nd
Graecumoside B	25.8	$C_{33}H_{48}O_{15}$	702.3331	0.1	$[M+NH_4]^+$	0.108	0.108	Nd	Nd	Nd	Nd	Nd	Nd	Nd	Nd	Nd

Dihydroxyflavone (daidzein or isomer)	26.7	$C_{15}H_{10}O_4$	255.0649	1.1	$[M + H]^+$	Nd	Nd	1.119	2.765			Nd			Nd	Nd
2″-*O*-*p*-Coumaroylvitexin	27.0	$C_{30}H_{26}O_{12}$	579.1483	2.4	$[M + H]^+$	2.387	2.077	0.177	1.766	Nd	Nd	Nd	Nd	Nd	3.130	Nd
1-Octen-3-ol, pentosyl hexoside	28.4	$C_{19}H_{34}O_{10}$	440.2484	1.4	$[M+NH_4]^+$	Nd	Nd	1.460	1.596	Nd	Nd	2.414	Nd	2.414	Nd	1.869
1-Octen-3-ol, pentosyl hexoside	29.1	$C_{19}H_{34}O_{10}$	440.2488	0.5	$[M+NH_4]^+$	Nd	Nd	0.551	0.483	Nd	Nd	1.801	Nd	0.824	Nd	Nd
1-Octen-3-ol, pentosyl hexoside	29.4	$C_{19}H_{34}O_{10}$	440.2486	1.0	$[M+NH_4]^+$	Nd	Nd	0.892	1.256	Nd	Nd	2.210	Nd	0.960	Nd	Nd
2″-*O*-*p*-Coumaroyl-orientin	30.7	$C_{30}H_{26}O_{13}$	595.1444	0.4	$[M + H]^+$	0.348	0.651	Nd	Nd	Nd	Nd	Nd	Nd	Nd	Nd	Nd
1-Octanol, pentosyl hexoside	31.0	$C_{19}H_{36}O_{10}$	442.2643	0.8	$[M+NH_4]^+$	Nd	Nd	0.820	1.024	Nd	Nd	Nd	Nd	Nd	Nd	Nd
Formononetin acetyl-hexoside	31.8	$C_{24}H_{24}O_{10}$	473.1433	2.0	$[M + H]^+$					Nd	Nd	Nd	Nd	2.057	Nd	Nd
Apigenin	32.6	$C_{15}H_{10}O_5$	271.0598	1.1	$[M + H]^+$	Nd	Nd	1.402	0.959	Nd	Nd	2.176	Nd	2.066	Nd	Nd
Trigraecum	35.3	$C_{16}H_{12}O_4$	269.0802	2.4	$[M + H]^+$	Nd	Nd	Nd	Nd	Nd	2.250	Nd	Nd	2.584	Nd	1.692
Medicarpin	36.2	$C_{16}H_{14}O_4$	271.0959	2.2	$[M + H]^+$	Nd	Nd	Nd	Nd	Nd	2.196	1.864	Nd	1.864	Nd	1.422
Trigoneoside XIIa or XIIb or trigofoenoside A	36.5	$C_{45}H_{74}O_{18}$	903.49414	0.7	$[M + H]^+$	Nd	Nd	Nd	Nd	Nd	Nd	0.721	Nd	Nd	Nd	Nd
2'-*O*-Methylvestitol	36.8	$C_{17}H_{18}O_4$	287.1275	1.0	$[M + H]^+$	Nd	Nd	Nd	Nd	Nd	2.039	Nd	Nd	2.457	Nd	Nd
Spirost-5-en-3-ol; (3β,25*R*)-form, 3-*O*-[α-L-rhamnopyranosyl-(1→3)-α-L-rhamnopyranosyl-(1→4)-β-D-glucopyranosyl-(1→4)-β-D-glucopyranoside]	40.5	$C_{51}H_{82}O_{21}$	1031.5396	2.5	$[M + H]^+$	0.334	0.490	1.324	0.101	Nd	Nd	2.507	Nd	Nd	Nd	Nd
Graecunin G or *Trigonella* saponin A or isomer	41.3	$C_{45}H_{72}O_{17}$	885.4836	0.7	$[M + H]^+$	0.444	0.866	1.487	0.245	0.177	Nd	1.837	Nd	Nd	0.663	Nd
Graecunin G or *Trigonella* saponin A or isomer	42.0	$C_{45}H_{72}O_{17}$	885.4822	2.3	$[M + H]^+$	0.595	0.094	0.731	0.173	Nd	Nd	2.244	Nd	Nd	Nd	Nd
Trigoneoside XIIa or XIIb or trigofoenoside A	45.5	$C_{45}H_{74}O_{18}$	920.5208	0.6	$[M+NH_4]^+$	Nd	Nd	Nd	Nd	Nd	Nd	Nd	Nd	Nd	0.577	Nd
Fenugreek saponin I or isomer	52.4	$C_{38}H_{62}O_{13}$	727.4256	1.0	$[M + H]^+$	0.149	0.099	1.496	0.575	Nd	Nd	1.661	0.987	1.331	Nd	Nd
Graecunin G or *Trigonella* saponin A or isomer	54.1	$C_{45}H_{72}O_{17}$	885.4836	0.7	$[M + H]^+$	Nd	0.173	0.026	0.997	Nd	Nd	0.663	Nd	Nd	Nd	Nd

All compounds assigned by comparison of accurate mass data (based on ppm#) and by interpretation of available MS/MS and/or UV spectra. Nd: Not detected/below the level of detection.

*Neocorymboside (occurs in *Trigonella* species), schaftoside, isoschaftoside, neoschaftoside, neoisoschaftoside, or isomer.

Sprout samples: 1st Methanol Extraction [1st FPME], 2nd Chloroform Extraction [2nd FPCE], 3rd Hexane Extraction [3rd FPHE], 1st Hexane Extraction [1st FPHE], 2nd Chloroform Extraction [2nd FPCE], 3rd Methanol Extraction [3rd FPME], (Seeds samples) 1st Methanol Extraction [1st FSME], 2nd Chloroform Extraction [2nd FSCE], 1st Hexane Extraction [1st FSHE], 2nd Chloroform Extraction [2nd FSCE], 3rd Methanol Extraction [3rd FSM].
